# Biodegradation of Pristine and Post-Consumer Extruded Expanded Polystyrene Packaging by *Zophobas atratus* Larvae: Influence of the Larval Stage and Physiological Response

**DOI:** 10.3390/polym17212870

**Published:** 2025-10-28

**Authors:** Juraci Duarte Pereira, Jamille Santos Santana, Paulo Vitor França Lemos, Denilson de Jesus Assis, Carolina Oliveira de Souza, Lucas Guimarães Cardoso, Alessandra Almeida Lucas, Lívia Maria Garcia Gonçalves, Rita de Cássia de Oliveira Sebastião, Bárbara Darós de Lelis Ferreira, Maria Betânia de Freitas Marques, Andrea Rebouças Rocha, Renata Quartieri Nascimento, Jania Betania Alves da Silva

**Affiliations:** 1 Graduate Program in Chemical Engineering (PPEQ), Polytechnic School, Federal University of Bahia (UFBA), Salvador 40210-630, BA, Brazil; juraci.duarte@hotmail.com (J.D.P.); denilson.assis@unifacs.br (D.d.J.A.); guimaraes.lucas@animaeducacao.com.br (L.G.C.); 2 State University of Bahia (UNEB), Salvador 41150-000, BA, Brazil; milepct@hotmail.com (J.S.S.); rqntri@gmail.com (R.Q.N.); 3 Institute of Pharmacy, Martin Luther University Halle-Wittenberg, Kurt-Mothes-Straße 3, 06120 Halle, Germany; paulo.lemos@pharmazie.uni-halle.de; 4 School of Exact and Technological Sciences, University Salvador (UNIFACS), Salvador 41820-021, BA, Brazil; 5 Department of Food Science, College of Pharmacy, Federal University of Bahia (UFBA), Salvador 40170-115, BA, Brazil; carolods@ufba.br; 6 Graduate Program in Food Science, College of Pharmacy, Federal University of Bahia (UFBA), Salvador 40170-115, BA, Brazil; andrea.engal@gmail.com; 7 Department of Materials Engineering, Federal University of São Carlos (UFSCAR), São Carlos 13565-905, SP, Brazil; alucas@ufscar.br (A.A.L.); liviagarciagoncalves@gmail.com (L.M.G.G.); 8 Department of Chemistry, Institute of Exact Sciences, Federal University of Minas Gerais (UFMG), Belo Horizonte 31270-901, MG, Brazil; ritacos@ufmg.br (R.d.C.d.O.S.); darosbdlf@gmail.com (B.D.d.L.F.); 9 Departament of Food and Drugs, Federal University of Alfenas (UNIFAL), Alfenas 37130-001, MG, Brazil; maria.marques@unifal-mg.edu.br; 10 Center for Exact and Technological Sciences, Federal University of Recôncavo da Bahia (UFRB), Cruz das Almas 44380-000, BA, Brazil

**Keywords:** superworms, bioremediation, white pollutant, physiological response, frass

## Abstract

Plastics are inexpensive and widely used but persist in the environment due to improper disposal. Insect-mediated biodegradation has gained attention, notably involving *Tenebrio molitor* larvae. Despite morphological similarities and larger size, *Zophobas atratus* larvae remain less studied. This work evaluated the impact of larval stage on the biodegradation of pristine and post-consumer extruded polystyrene (XPS) and the physiological effects of an XPS-based diet. Smaller (L1) and larger (L2) larvae were tested. L2 showed higher XPS consumption, weight gain, and survival, while XPS-fed larvae overall exhibited reduced lipid content and increased moisture, flavonoids, and phenolics compared to wheat bran-fed controls. Scanning electron microscopy revealed surface fragmentation in frass, more pronounced in L1, suggesting greater mechanical or enzymatic action. High-performance size exclusion chromatography indicated molecular weight reduction, with L1 more effective on pristine XPS and L2 on post-consumer XPS, likely due to nutritional residues. FTIR analysis showed oxidative changes in both groups, more prominent in L1. Thermogravimetric analysis revealed earlier degradation onset in L1 frass, supporting the presence of oxidized oligomers. Overall, *Z. atratus* larvae can biodegrade XPS, with degradation influenced by developmental stage and substrate type. These findings inform biotechnological strategies for sustainable plastic waste management.

## 1. Introduction

Global population growth has driven the development of materials whose waste, when improperly disposed of, can remain in the ecosystem for hundreds of years [1,2,3]. According to the Brazilian Association of Waste and Environment (ABREMA), dry materials represent up to 33.6% of the total 81 million tons of municipal solid waste (MSW). These materials are mainly composed of plastics (13.8 million tons per year, 16.8% of MSW), with only 8% of the dry waste recycled or processed to develop new materials [4]. Extruded expanded polystyrene (XPS) is one of the plastic materials present in this waste, which has a negative impact on the environment. According to Plastics Europe, the global production of polystyrene in its rigid form and XPS as a foam has reached 21 million tons [5].

Improperly disposed extruded expanded polystyrene (XPS) fragments endure for decades in terrestrial and aquatic ecosystems, where they disintegrate into micro- and nanoplastics that are readily consumed by fauna and accumulate in sediments and food webs [6,7]. These particles can block the intestines, cause oxidative stress, and slow down the growth and feeding of aquatic animals and soil invertebrates. They can also change the structure of the soil and how well it holds water, which can hurt photosynthesis and root growth in plants [7]. Such impacts emphasize the need to quickly develop environmentally friendly degradation pathways. Therefore, biological strategies that promote XPS biodegradation—such as those involving insect larvae—offer a sustainable and ecologically relevant alternative to mitigate the long-term persistence of this polymer in nature.

XPS is a type of foam produced through the expansion and extrusion process of polystyrene, which is commonly used in food packaging as well as in civil construction [8]. XPS is a durable plastic, but it is typically used for single-use applications, such as packaging [9,10,11]. XPS consumption in delivery orders increased by 46% between 2019 and 2021, which was mainly influenced by the SARS-CoV-2 pandemic [12].

XPS waste recovery mainly employs mechanical recycling, which includes crushing, compacting, and extruding the material to create threads and granules that can be reused to make new parts [13,14]. Other methods for the removal of XPS residue include burning it or dissolving the polymer using fluids that are in a supercritical state [15].

Various factors compromise the reverse logistics of XPS-based products. The challenges encountered in collecting materials include the low mass:volume ratio, low economic attractiveness, and presence of contamination and dirt, which discourages collectors and cooperatives [16]. The mismatch between the rapid production and recovery of XPS-based material waste substantially contributes to the increase in white pollutants in the environment. Plastic waste can later be degraded into small pieces, such as micro- and nano-plastics, through wear and tear, which can result in various forms of harm to nature and humans [12,17]. This imbalance highlights the urgent need for improved waste management and recycling processes of XPS materials. Addressing this issue is essential to reduce environmental pollution and promote sustainability.

Biodegradation can be one of the alternatives for controlling the improper disposal of plastics [18,19,20]. During this disintegration process, organisms alter the polymers’ molecular structure to produce compounds that may serve another function or application [21].

The use of insect larvae from the Tenebrionidae family for the biodegradation of plastics has been gaining prominence in the last decade [22,23]. Some examples of species in this family are *Tenebrio molitor* and *Zophobas atratus*. These insects exhibit four stages: egg, larva, pupa, and beetle [24]. Generally, insects in the larval stage are used to feed birds, fish, and other animals [25]. A characteristic of these larvae is the use of mandibular claws, which allows them to chew various residues, including polymers [26]. Recent studies have indicated that the larvae of *T. molitor* [27] and *T. obscurus* [28] can chew, break down, and mineralize XPS and polyethylene. Moreover, mealworms can be fed other types of plastic waste, such as polyvinyl chloride, polylactic acid, polyurethane, and polyethylene terephthalate [29,30,31], as well as vulcanized synthetic styrene/butadiene rubber and tire crumbs [32].

The mechanism for the degradation of plastics by *T. molitor* is dependent on the microbiota present in the larval intestine [27,33,34]. Recent studies have shown that when *T. molitor* larvae were given the antibiotic gentamicin, they could not break down and depolymerize polystyrene [9]. In addition to gentamicin, the antibiotic ampicillin and fungicide nystatin were evaluated in studies of polystyrene biodegradation by *T. molitor* larvae. Different degrees of polymer degradation were observed, with lower degradation values observed for larvae treated with nystatin [35]. This inability to break down plastic reveals that the manner in which the larvae digest plastic relies on their gut bacteria.

*Z. atratus* larvae are at least twice the size of *T. molitor*, chew more aggressively, and can break down plastics such as expanded polystyrene, polyvinyl chloride, polylactic acid, polypropylene, and polyethylene [36,37,38,39,40,41]. Therefore, we first hypothesized that *Z. atratus* at the larval stage could improve the efficiency of XPS biodegradation. Our second hypothesis was that the presence of food residues and dirt in XPS-based packing could improve the biodegradation by the larvae.

This study aimed to investigate the influence of the larval stage of *Z. atratus* on the biodegradation of pristine and post-consumer XPS-based packaging, as well as to evaluate the larvae’s physiological response to an XPS-based diet. We demonstrated the effect of the *Z. atratus* larval stage in degrading XPS control samples, mainly by using XPS-based packing, representing an environmentally relevant condition.

## 2. Materials and Methods

### 2.1. Larvae and Their Diets

*Z. atratus* larvae were purchased from SuperBugs-Alimentos Funcionais (Salvador, BA, Brazil), and commercial feed (wheat bran) was purchased from Carbrás Ltda (Lauro de Freitas, BA, Brazil). XPS was obtained in two ways: pristine XPS was purchased from a local store (Salvador Embalagens, Salvador, BA, Brazil), and post-consumer XPS was obtained from household waste consisting of disposable packaging that was used to store and transport food.

### 2.2. Biodegradation Experiments

The biodegradation experiment was performed according to a method adapted from Palmer et al. (2022) [42]. Two distinct groups of *Z. atratus* larvae were used: L1 (length between 0.5 and 3.0 cm) and L2 (between 5.0 and 7.0 cm). Initially, the larvae were subjected to 36 h of starvation to facilitate intestinal emptying. They were then separated into polypropylene boxes measuring 30 × 20 × 8 cm (length × height × width), with an opening at the top (7.5 × 15 cm), and covered with a screen (25 mesh) (Figure S1a–c). Following this, we subjected the two groups (L1 and L2) of larvae to three different diets, as detailed in Table 1. A total of 90 larvae were added to each box, and three independent replicates were performed for each diet (XPSLP, XPSPC, and RC) and each group of larvae (L1 and L2).

We provided the diets to the larvae in each box at a ratio of 2:1 (*w*/*w*) (Figure S1). The diet was replaced every 7 days only for the larvae that were fed commercial food to maintain the diet/larva ratio. The boxes were arranged on the shelves of a rack (Figure S2a–e) at a temperature of 26 ± 1 °C, with a relative humidity of 51 ± 4% (Minipa MT-241 digital thermo-hygrometer, São Paulo, SP, Brazil). We removed dead larvae and exoskeletons that resulted from skin shedding from the boxes during the experiment to prevent cannibalism. The larvae were hydrated using daily moistened gauze. The larvae and frass were counted and weighed every 15 days using an analytical balance (Shimadzu AY220, Kyoto, Japan). At the end of the experiment, the surviving larvae were starved for 36 h to empty their intestines and then frozen at −80 °C in an ultrafreezer (Coldlab, CL580-86V, São Paulo, SP, Brazil) for further analyses.

The physiological response of *Z. atratus* larvae to the RC, XPSLP, and XPSPC diets was quantified through the diet consumption rate, survival rate, and average larval weight. Diet consumption rate was calculated from the initial (*m_i_*) and final (*m_f_*) mass values of the diets (Equation (1)), as previously described by Bulak et al. (2021) [30].

(1) $$Diet\;utilization\;(\%)=\left(\frac{m_{i}-m_{f}}{m_{i}}\right)\times 100$$

We calculated the survival rate according to the description provided by Ding et al. (2024) [43]. Where no is the number of larvae at the beginning of the experiment and *n_i_* is the number of live larvae on each day of counting, according to Equation (2).

(2) $$Survival\;rate\;\left(\%\right)=\left(\frac{n_{i}}{n_{o}}\right)\times 100$$

The average weight (*g*) of the larvae was calculated during the experiment [43]. *P_t_* represents the total weight of all larvae per diet, and *N_t_* represents the number of live larvae per diet (Equation (3)).

(3) $$Average\;weight\;(g)=\left(\frac{P_{t}}{N_{t}}\right)$$

### 2.3. Physicochemical Composition of Larvae

#### 2.3.1. Total Moisture Content and Lipids

The AOAC method [44] was used to determine the physicochemical composition of the *Z. atratus* larvae. The moisture content was determined by drying in an oven at 105 °C (DeLeo, model DL-SE 1211, Brazil) until a constant weight was achieved. Lipids were extracted according to the Bligh and Dyer cold method, using a 2:1:1 mixture of chloroform, methanol, and water, respectively, and quantification by gravimetry [25,45].

#### 2.3.2. Analysis of the Fatty Acid Profile by Gas Chromatography

We identified and quantified the fatty acids in the diets and larvae using the method previously described by Souza et al. (2017) [46]. Transesterification of the total lipids was performed by adding 0.025 g of the sample to a methanolic solution of NaOH (0.5M) and boron trifluoride (BF_3_, 12% *m*/*v*). The resulting fatty acid methyl esters (FAMEs) were extracted using isooctane for chromatographic analysis.

FAME separation was performed using gas chromatography coupled with a flame ionization detector (Clarus 680, Perkin-Elmer, Springfield, IL, USA). The system was equipped with a DB-Fast FAME column (30 m × 0.25 mm × 0.25 µm). A 1 µL aliquot of the FAME solution was injected in the split mode (1:50 ratio) at 250 °C. Nitrogen was used as the carrier gas at a constant flow rate of 1 mL/min. The oven temperature program was as follows: an initial temperature of 60 °C for 0.5 min, increasing by 25 °C/min to 194 °C, remaining at this temperature for 1 min, increasing by 5 °C/min to 235 °C, and remaining at this temperature for 1 min. The detector temperature was 280 °C.

The identification of fatty acids was performed by comparing the retention time of the sample peaks with the methyl esters of the fatty acids in a standard mixture (C4–C24, 189–19, Sigma-Aldrich, St. Louis, MO, USA). Quantification was performed based on the normalization of the peak areas. The indices of the saturated fatty acids (SFA), total monounsaturated fatty acids (MUFA), and polyunsaturated fatty acids (PUFA) were calculated based on the identified and normalized fatty acids.

#### 2.3.3. Analysis of Phenolic and Flavonoid Compounds

Phenolic and flavonoid compounds were determined in the macerated and lyophilized larvae. The Folin–Ciocalteu method [47], was employed to quantify the total phenolic compounds. The polyphenolic extracts were measured using a spectrophotometer (Lambda 35, Perkin-Elmer, USA) at a wavelength of 762 nm. To measure the amount, a standard curve was made with 20–100 mg/L of gallic acid (Sigma-Aldrich), and the results were presented in mg of gallic acid equivalent per 100 g of the sample (mg GAE/100 g).

Flavonoids were determined using the previously described method of Lee et al. (2003) [48]. The polyphenolic extracts were analyzed with a spectrophotometer (Lambda 35, Perkin-Elmer, USA) at a wavelength of 510 nm. Quantification was performed using a standard curve (0.1–1 mg/mL) of epicatechin (Sigma-Aldrich), and the results were presented in mg of epicatechin equivalent per 100 g of sample (mg EPI/100 g).

### 2.4. Characterization of the Diets and Frass

#### 2.4.1. Scanning Electron Microscopy (SEM)

The samples were carefully deposited onto carbon tape and coated with a layer of gold (Bal-tec Cool Sputter Coater, SDC 005). The tiny structures of the diets and frass were examined using SEM (Jeol, model JSM-6610LV, Tokyo, Japan) at 500× magnification, with a clarity of 50 µm, and a voltage set to 5 kV [30].

#### 2.4.2. High Performance Size Exclusion Chromatography (HPSEC)

HPSEC was used to determine the average molecular mass of XPS and the leftover polymer in the larval waste. The XPS samples (XPSLP and XPSPC) and the corresponding frass were weighed and dissolved in 20 mL of tetrahydrofuran at a temperature of 25 ± 2 °C for 12 h. The solution was filtered and placed on a magnetic stirrer at 60 °C until the volume was reduced to 5 mL by evaporation.

A 100 µL aliquot was placed in an autosampler and then injected into a chromatograph (Varian PL-GPC 50 Agilent, Santa Clara, CA, USA). Separation was performed with the SEC columns Shodex KF805, KF804, and KF804 connected in series at a constant flow of tetrahydrofuran at 1.0 mL/min and a steady temperature of 40 °C [49]. The eluates were detected with a refractive index detector (Viscotek 350B, Malvern, PA, USA).

The molecular weights (Mw) of the XPS polymers were estimated by constructing an external analytical curve. The analytical curve was successfully constructed using 10 polystyrene standards (American Polymer Standards) that were prepared in the same manner as the samples and then injected. The logarithm of the standard Mw and average retention time of each sample measured in triplicate (SD < 0.05) were highly correlated and provided a linear fit (R^2^ = 0.9904).

The polydispersity index (*PDI*) was calculated from the relationship between the weight-average molar mass (*Mw*) and the number-average molar mass (*Mn*), according to Equation (4).

(4) $$PDI=\left(\frac{Mw}{Mn}\right)$$

#### 2.4.3. Fourier Transform Infrared Spectrometry with Attenuated Total Reflectance (FTIR-ATR)

The infrared spectra were recorded using a Spectrum Two spectrometer (Perkin-Elmer, USA) equipped with an attenuated total reflectance device featuring a ZnSe crystal. Data acquisition involved 64 scans accumulated at room temperature (25 °C), with a spectral resolution of 2 cm^−1^ [50].

#### 2.4.4. Thermogravimetric Analysis (TGA)

The samples were analyzed via thermogravimetry using a DTG60H thermogravimetric analyzer (Shimadzu, Kyoto, Japan). Approximately 2.5 mg of the sample was weighed in alumina crucibles. Analyses were performed in an N_2_ atmosphere with a constant flow of 50 mL/min and a heating rate of 10 °C/min from 30 to 500 °C. The first derivative curves (DTG), onset temperature of thermal decomposition T_onset_ events, and mass loss were calculated using the manufacturer’s software and OriginLab 8.5 software.

#### 2.4.5. Statistical Analysis

The experimental design was completely randomized. The analyses were performed in triplicate (*n* = 3) for each experimental group, and the results were expressed as the arithmetic mean ± standard deviation (SD).

## 3. Results and Discussion

### 3.1. Physiological Response of Z. atratus Larvae

**Figure 1:**
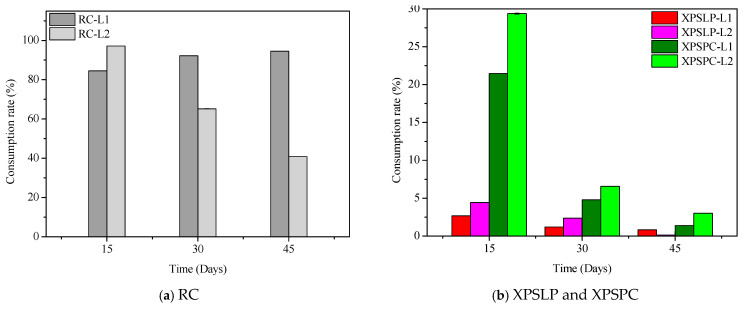
Consumption and survival rates of *Zophobas atratus* larvae (L1 and L2) fed with pristine (XPSLP) and post-consumer (XPSPC) XPS packaging. Conventional diet (RC) and XPS-based diet (XPSLP and XPSPC). Data represent mean ± SD (*n* = 3).

**Figure 2:**
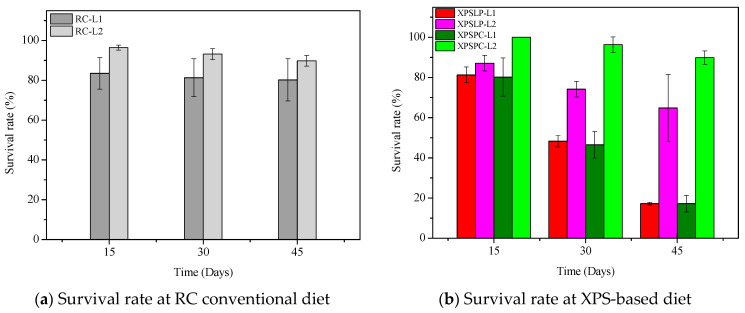

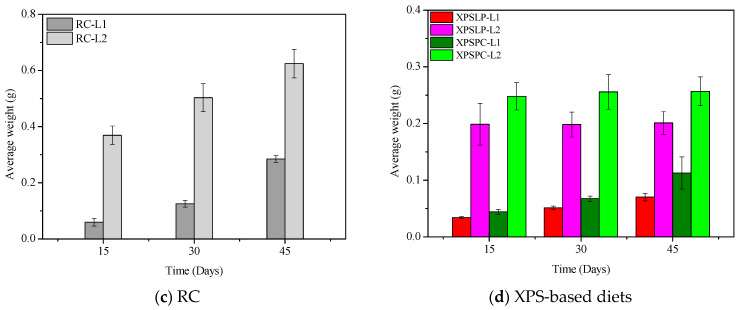
Average weight of *Z. atratus* larvae (L1 and L2) fed with control (RC), pristine (XPSLP), and post-consumer (XPSPC) diets. Data represent mean ± SD (*n* = 3).

The diet consumption and survival rates of *Zophobas atratus* larvae (L1 and L2) are summarized in Figure 1 and Table S1. Both parameters followed similar trends: larger larvae (L2) consumed more XPS and maintained higher survival throughout the 45-day experiment than smaller larvae (L1), particularly when fed with post-consumer packaging (XPSPC). In contrast, L1 larvae showed lower consumption and survival when reared on pristine XPS (XPSLP). These results indicate that substrate contamination and developmental stage strongly influence feeding behavior and tolerance to XPS-based diets, consistent with the nutritional limitations of polymer-derived substrates previously reported for *Tenebrio molitor* [37,39,41]. The evolution of larval weight under different diets is presented in Figure 2, confirming that L2 larvae achieved greater biomass gain than L1, especially when exposed to post-consumer XPS. Overall, our findings demonstrate that *Z. atratus* larvae—particularly at advanced stages—can biodegrade both pristine and post-consumer XPS under minimal pre-treatment, supporting their potential use in sustainable plastic-waste management systems.

In contrast to previous studies focusing on *Tenebrio molitor* and *Zophobas morio*, which mainly examined polystyrene degradation under pristine laboratory conditions, our findings reveal that *Zophobas atratus* larvae can biodegrade both pristine and post-consumer XPS packaging. Moreover, this study provides the first evidence that the larval developmental stage strongly modulates biodegradation performance—smaller larvae (L1) enhanced polymer oxidation and molecular breakdown, whereas larger larvae (L2) exhibited greater intake, survival, and adaptation when exposed to post-consumer XPS. This dual influence of substrate contamination and developmental stage represents a novel aspect of XPS biodegradation among Tenebrionidae species.

From a practical and economic perspective, growing *Zophobas atratus* larvae for XPS biodegradation has a number of benefits that could make this method possible on a larger scale. The species is easy to raise, does not need much infrastructure, and can live on cheap agro-industrial byproducts or organic waste, which keeps feed and operating expenses low. Furthermore, larvae are already being sold as animal feed, which means that there are already big production systems and supply chains in place. When added to current waste management systems, larval processing could be a cheap biological pretreatment step that makes polystyrene waste smaller and lighter, making it easier for microbes or chemicals to break it down further. These traits make *Z. atratus*-based biodegradation a beneficial choice for dealing with plastics after they have been used because it is good for the environment and costs less.

### 3.2. Physicochemical Composition of the Larvae

The moisture, lipids, fatty acids, phenolic, and flavonoid contents of the larvae are presented in Table 2. The final mass of the larvae in group L1 was not sufficient to perform physicochemical composition analyses.

XPSPC-L2-fed larvae exhibited the highest moisture content (78.12%), followed by XPSLP-L2 (67.77%) and RC-L2 (56.23%). These values exceed those reported by Peng et al. (2023) [49] for *T. molitor* fed polyethylene (59.1%) and flounder meal (62.2%) diets, which suggests that polymer mineralization during biodegradation may contribute to increased moisture. This is because mineralization releases H_2_O as a byproduct of microbial respiration, thereby intrinsically elevating the moisture content [51].

The lipid content for the XPS, XPSLP-L2 (3.47%), and XPSPC-L2 (2.96%) diets was lower than that of the RC-L2 control diet (15.30%). According to Bożek et al. (2017) [52], substantial changes in lipid levels were observed during stress and starvation. Larvae subjected to XPS diets underwent food stress during the process of adaptation to the polymeric diet, which may have resulted in the metabolization of their body mass. Our findings match the reduction in fat levels, which are a source of energy for *Z. atratus* larvae.

The four types of fatty acids were identified. We observed two SFA (palmitic acid—C16:0 and stearic acid—C18:0) and two unsaturated fatty acids (monounsaturated oleic acid—C18:1ω-9 and polyunsaturated linoleic acid—C18:2 ω-6, [53]. RC-L2 showed a higher palmitic acid content (37.11%) compared to XPSLP-L2 (33.44%) and XPSPC-L2 (33.53%). Nascimento et al. (2022) [25] found that larvae fed with commercial feed (RC) had 34.37% palmitic acid, which dropped to 28.22% when they were fed a diet with grape leftovers. The stearic acid content of XPSLP-L2 (10.37%) and XPSPC-L2 (10.31%) was higher than that of RC-L2 (8.16%). The same pattern was observed for oleic acid, with RC-L2 (30.61%), XPSLP-L2 (31.82%), and XPSPC-L2 (33.31%). The linoleic acid content was the same for RC-L2 (22.25%) and XPSLP-L2 (22.25%) and lower for XPSPC-L2 (20.79%).

The stearic, oleic, and linoleic acid levels were consistent with those reported by Nascimento et al. (2022) [25], indicating a minimal impact of the XPS diet on these fatty acids. Slight variations observed may result from reduced lipid content due to food stress, prompting the mobilization of stored lipids for energy [24,52]. This lipid mobilization, while providing a temporary energy source, also leads to a reduction in larval body mass, which in turn diminishes their physiological capacity to sustain efficient polymer biodegradation over time.

The phenolic compound contents in the larvae fed the experimental diets were 5.59% (XPSLP-L2), 1.72% (XPSPC-L2), and 3.26% (RC-L2). Flavonoids, as a subclass of phenolic compounds, followed a similar trend, with concentrations of 0.040 mg/100 g (XPSLP-L2), 0.041 mg/100 g (XPSPC-L2), and 0.012 mg/100 g (RC-L2). These compounds may be acquired as larvae metabolize the ingested materials, particularly those with specific chemical structures. However, as reported by Nascimento et al. (2022) [25], elevated phenolic levels can increase insect mortality by forming complexes with proteins, thereby impairing digestion and nutrient absorption. This mechanism may explain the reduced consumption observed for XPS-based diets and lower survival rates observed at the end of the experiment.

### 3.3. Characterization of Larval Frass

#### 3.3.1. Scanning Electron Microscopy

**Figure 3:**
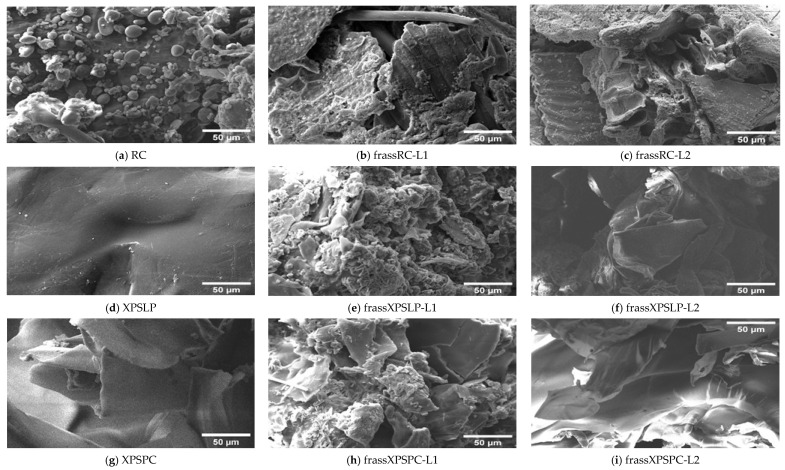
Micrographs of the RC, XPSLP, and XPSPC diets and the respective frass generated by larvae of groups L1 and L2. (**a**) RC, (**b**) frassRC-L1, (**c**) frassRC-L2, (**d**) XPSLP, (**e**) frassXPSLP-L1, (**f**) frassXPSLP-L2, (**g**) XPSPC, (**h**) frassXPSPC-L1, (**i**) frassXPSPC-L2.

Differences in the morphology of the diets and their respective frasses were observed using SEM. We observed grain-like shapes in the RC (Figure 3a). In contrast, the frass from RC-L1 and RC-L2 showed overlapping plates with raised areas (Figure 3b,c). A smooth surface with small grooves and longitudinal cracks was observed in the XPSLP (Figure 3d). However, the frass from XPSLP-L1 and XPSLP-L2 (Figure 3e,f) presented an irregularly sculpted and fragmented structure, which may be an indication of biodegradation by *Z. atratus* larvae.

The XPSLP-L2 frass exhibits a morphology similar to that of XPSLP, which is characterized by smooth surface plates. However, frass from XPSLP-L1 has a broken structure, which may indicate that L1 larvae digest food more effectively. Bulak et al. (2021) [30] examined the morphology of frass produced by *T. molitor* larvae fed a polystyrene diet and reported results analogous to those of *Z. atratus* larvae in this study.

Despite having a smooth surface, XPSPC exhibits fragmentation and edges (Figure 3g). XPSPC had contact with the feed, which may have promoted changes in the surface structure of the polymer by abrasion [49]. The frass samples from XPSPC-L1 and XPSPC-L2 (Figure 3h,i) presented a structure similar to that of frass from XPSLP-L1. However, the frass samples exhibited plaques similar to the XPSPC diet, which indicates no morphological difference when fed with XPS. In other words, the presence of food residues in the XPSPC packages did not alter the micrometric morphology of the frass generated by the larvae of groups L1 and L2. However, the XPS frass generated by the smaller larvae of group L1 exhibited a more heterogeneous and discontinuous surface.

Overall, the SEM analysis revealed morphological features that are consistent with the partial biodegradation of the diets by *Z. atratus* larvae. While similarities were observed between frass and their respective diets, particularly from the XPSPC samples, distinct surface alterations such as fragmentation, irregular sculpting, and structural discontinuities were more pronounced in frass from group L1, which suggests more active physical or enzymatic breakdown by the smaller larvae. Notably, frass derived from the XPSLP and XPSPC diets retained some characteristics of the original materials, but signs of surface disruption indicate mechanical processing during digestion. The greater efficiency of L1 larvae, despite their lower consumption, likely reflects their faster metabolism, higher enzymatic activity, and more active gut microbiota, which together enhances the digestion and biodegradation of polymers at early developmental stages [54,55].

These observations support the hypothesis of limited yet measurable biodegradation of XPS-based materials by *Z. atratus*, with the larval group and diet composition influencing the degree of morphological alteration.

#### 3.3.2. High Performance Size Exclusion Chromatography

We used chromatographic analysis to check how biodegradation changed the molecular mass distribution of XPS recovered in the frass produced by the larvae from groups L1 and L2.

**Figure 4:**
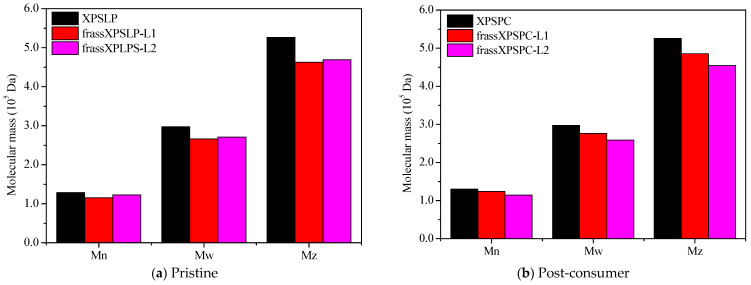
HPSEC. A comparison of M_w_, M_n_, and M_z_ for (**a**) pristine and (**b**) post-consumer XPS (XPSPC)-based packaging. The XPSLP and XPSPC samples are compared with the XPS-recovered larvae’s frass.

Changes in the values of the numerical average molecular mass (Mn), weight average molar mass (Mw), Z average molar mass (Mz), and polydispersity index (PDI) are considered key signs of changes, breakdown, and deterioration of XPS [9]. The measurements taken from the larvae show that the XPS diet was partly broken down, as the waste still had the polymer in it (Figure 4).

XPSPC and its corresponding frass samples from XPSPC-L1 and XPSPC-L2 exhibited reductions in the Mn, Mw, and Mz values, following trends similar to those observed for XPSLP (Figure 4b and Table S2). However, contrary to the XPSLP results, group L2 showed more pronounced reductions. In group L1, Mn decreased by 4.70% (from 130,173 to 124,049 Da), while in group L2, it dropped by 12.10% (from 130,173 to 114,427 Da). The Mw decreased by 7.11% in the frass from XPSPC-L1 (from 297,176 to 276,033 Da) and by 12.93% in the frass from XPSPC-L2 (from 297,176 to 258,747 Da). Similarly, Mz was reduced by 7.61% in group L1 (from 525,558 to 485,562 Da) and by 13.57% in group L2 (from 525,558 to 454,218 Da). The PDI decreased in both frass samples, reaching 2.23 in group L1 (−2.19%) and 2.26 in group L2 (−0.87%).

These results indicate that XPSPC underwent more substantial molecular weight reductions when processed by the group L2 larvae, whereas XPSLP showed greater degradation by the group L1 larvae. The presence of food residues in the XPSPC diet likely enhanced the polymer chain breakdown during digestion by *Z. atratus*, and the effect was more pronounced in the more developed L2 larvae group. This suggests that the nutritional composition of the substrate and larval stage play critical roles in the extent of XPS degradation.

#### 3.3.3. Fourier Transform Infrared Spectrometry with Attenuated Total Reflectance

FTIR revealed chemical modifications in the XPS polymer structure, thus demonstrating biodegradation in the larvae’s gut.

**Figure 5:**
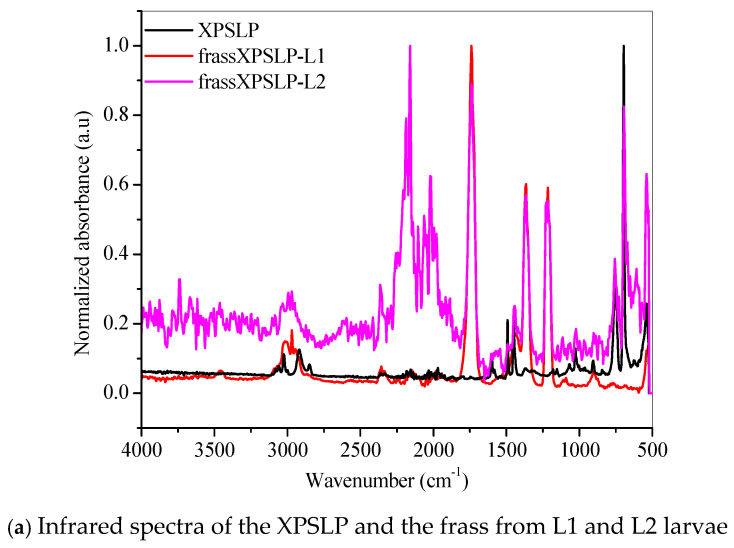

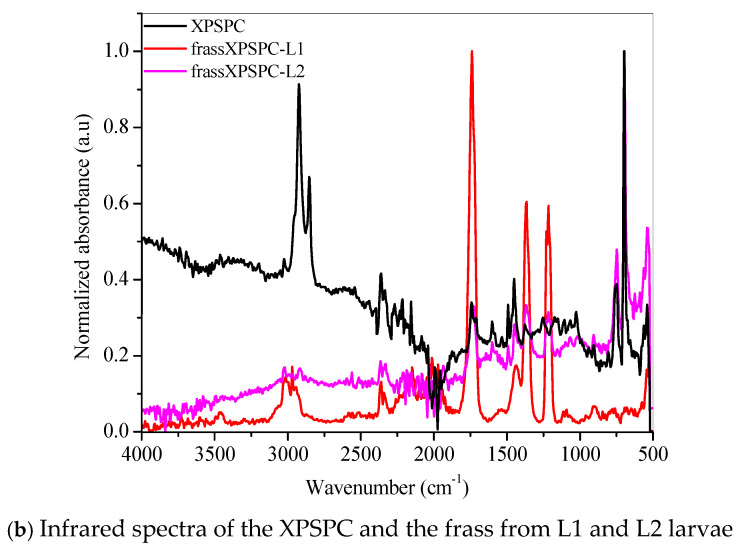
FTIR spectra of the (**a**) pristine XPS packaging (XPSLP) and (**b**) used packaging (XPSPC), and the corresponding frass from larvae (L1 and L2) are presented.

The FTIR spectrum of the XPSLP sample showed typical absorption bands from the XPS polymer (Figure 5a and Figure S3). In the region between 3100 cm^−1^ and 3000 cm^−1^, four distinct bands were observed with very low intensity, which are attributed to the stretching vibrations of hydroxyl (–OH) groups [56]. Two prominent absorption bands were identified at 2917 cm^−1^ and 2848 cm^−1^, which correspond to the asymmetric and symmetric stretching vibrations of the C–H bonds, respectively [56]. In addition, a spectral band centered at 1492 cm^−1^ was associated with the stretching of the aromatic ring, while another band at 1452 cm^−1^ was attributed to the CH_2_ bending vibrations. Finally, a band near 700 cm^−1^ was observed, which corresponds to out-of-plane C–H bending, a typical feature of the aromatic compounds present in the XPS polymer structure [56].

Evidence of biodegradation can be seen in Figure 5a. The infrared spectra of the frass from XPSLP-L1 and the frass from XPSLP-L2 showed strong absorptions centered at 1739 cm^−1^, 1365 cm^−1^, and 1214 cm^−1^, which were not observed in the spectrum of the pristine XPS packaging (black line). The vibrations of the ester carbonyl group (1739 cm^−1^), –COH bending (1365 cm^−1^) [57], and –COH stretching (1214 cm^−1^) [39,57] indicate the oxidation of the XPS. The reactions biocatalyzed by the larval gut microbiota resulted in XPS oligomers, as revealed by the presence of ester and aldehyde groups. Our observations corroborate the ability of *Z. atratus* larvae to produce enzymes capable of digesting plastics, as reported by Wang et al. (2024) [58].

The fingerprint region showed an even more intriguing difference (Figure 5a). The well-defined spectral band centered at 696 cm^−1^, which is typical of an out-of-plane C–H bending that originates from an aromatic ring [56], was preserved in the frass from the XPSLP-L2 sample but not in the frass from XPSLP-L1. This spectral difference confirms the influence of the larval stage on the biodegradation of plastics. The absence of this specific vibrational feature at 696 cm^−1^ in the frass from the XPSLP-L1 sample indicates that the plastic’s chemical structure has been significantly changed due to the larvae’s activity. Consequently, this finding underscores how different larval stages can uniquely impact the degradation process.

Evidence of biodegradation can also be seen in Figure 5b. The infrared spectra of XPSPC, including the frass from XPSPC-L1 and XPSPC-L2, followed the same trend observed in the group of pristine XPS packaging discussed in Figure 5a.

Our results indicate that biodegradation in the larval gut led to oxidative processes in both L1 and L2 groups. According to the infrared spectra, the smaller larval group (L1) demonstrated a greater capacity for XPS biodegradation, regardless of whether the packaging was new or used. These findings suggest that L1 larvae are more effective at breaking down XPS, which could make them more suitable for waste management applications.

#### 3.3.4. Thermogravimetry

**Figure 6:**
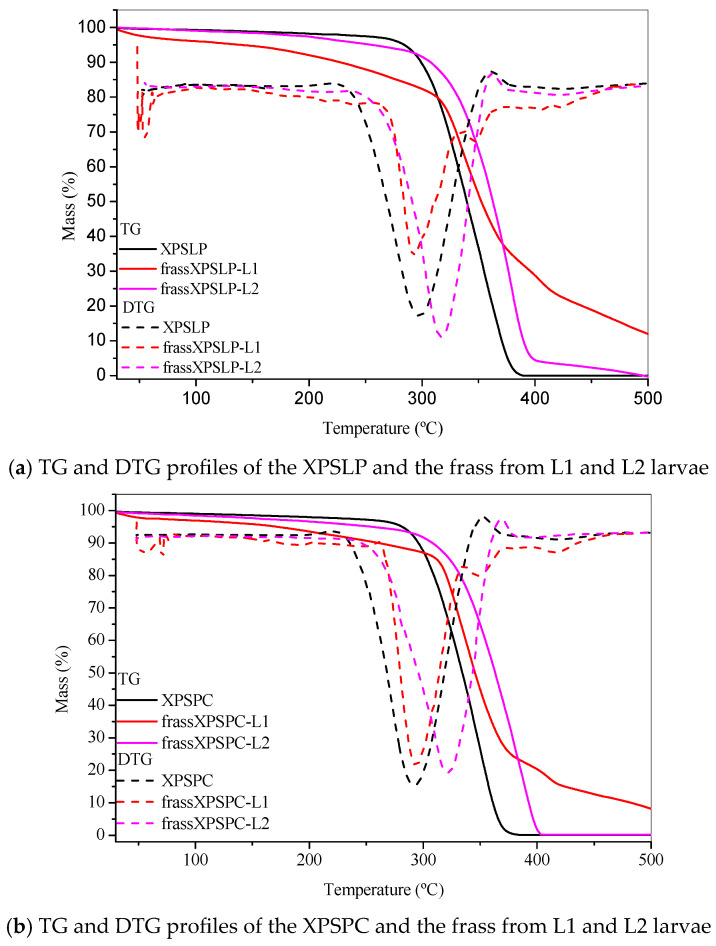
Thermogravimetry. TG and DTG profiles of the (**a**) pristine XPS packaging (XPSLP) and (**b**) used packaging (XPSPC), and the corresponding frass from larvae (L1 and L2) are presented.

The thermogravimetry profiles of the pristine, unused XPS packaging sample, which was used as a control (XPSLP), revealed a characteristic thermal decomposition profile (Figure 6a). The thermogravimetry curve shows a negligible mass loss of <2% between 25 °C and 150 °C, indicating minimal moisture or volatile content in the sample. The Tonset DTG of XPSLP (244 °C) (Figure 6a) was lower than those commonly reported for polystyrene used in the food industry (~280 °C, [59]). This difference was due to the way the material was processed [60] and additives that were used to change the XPS properties, such as plasticizers or expanding agents, which can make the material less thermally stable [61].

The T_onset_ DTG was 244 °C, which is consistent with the degradation behavior of polystyrene. At 500 °C, the XPSLP sample exhibited nearly complete thermal degradation, with a residual mass close to 0%, thus confirming the predominantly organic nature of the material.

The thermogravimetry and DTG profiles of XPSLP and frass from XPSLP-L2 were similar in shape and T_onset_, which was in contrast to the frass from the XPSLP-L1 sample (Figure 6a). The thermogravimetry profile of the smaller larvae in the L1 group showed approximately 6% mass loss between 30 °C and 150 °C (Figure 6a), indicating that low-molecular-weight and low-vapor-pressure compounds were released according to XPS. This mass loss might be due to byproducts from biodegradation that are attached to the polymer structure, such as water, organic acids, and aldehydes.

The DTG curve of the frass from the XPSLP-L1 sample had a bump at Tonset 146 °C, indicating that it started to break down earlier compared to XPSLP. The early breakdown seen mostly in the waste from the smaller larvae of group L1 matches the heat stability of the oxidized XPS oligomers, as shown by HPSEC and FTIR-ATR.

Considerable differences were also observed in the residual masses of the samples at 500 °C, as shown in Figure 6a. While the residues of XPSLP and the frass from XPSLP-L2 were approximately 0%, the frass from the XPSLP-L1 sample exhibited a markedly higher residue of 12%. This discrepancy suggests the more effective physical processing of the XPS matrix by the group L1 larvae; however, the presence of a higher residue also indicates that the ingested polymer was not fully mineralized. Instead, a significant portion of the XPS remained as thermally stable fragments, which implies that the material was primarily fragmented and only partially chemically altered. Therefore, the elevated residue highlights that the biodegradation process was incomplete, with the polymer chains undergoing limited depolymerization and mineralization while persisting as solid byproducts in the frass.

The TG and DTG profiles of XPSPC and the frass from XPSPC-L1 and XPSPC-L2 (Figure 6b) exhibited the same pattern as the curves in Figure 6a, which indicates that they also showed signs of biodegradation.

While microbiota profiling and enzyme assays were not conducted in this study, an increasing body of evidence suggests that the degradation of polystyrene (PS/EPS/XPS) by *Zophobas atratus* is significantly influenced by gut microorganisms and their associated enzymatic pathways.

In *Z. atratus* larvae fed PS or EPS, alterations in the gut microbial community have been documented, characterized by the enrichment of Pseudomonas strains isolated from the larval gut that degrade PS in vitro, alongside the up-regulation of serine hydrolase genes and the surface oxidation of PS (e.g., an increase in carbonyl groups and a transition from a hydrophobic to a hydrophilic surface) [62]. Gut bacterial consortia derived from Z. atratus have been enriched and demonstrated the ability to degrade PS, suggesting metabolic pathways such as styrene to phenylacetyl-CoA, in alignment with oxidation and ring-cleavage mechanisms [63]. Simultaneously, feeding larvae plastic diets alters the activities of digestive enzymes (e.g., oxidases and hydrolases) in the insect gut, highlighting the potential for insect–microbiome synergistic enzymatic degradation [64].

Based on these findings, we interpret our observations of XPS mass loss, surface oxidation, and chemical modifications in our samples as plausibly resulting from microbiota-assisted enzymatic degradation.

## 4. Conclusions

*Z. atratus* larvae at different developmental stages can consume only XPS packaging as a diet for at least 45 days. The results showed that larvae are able to consume more used XPS packaging than unused packaging. The most developed larvae (group L2) presented higher survival rates, diet consumption, and weight gain. The lipid content of larvae provided with a diet based on XPS packaging decreased in relation to the conventional wheat bran diet. However, there was an increase in the moisture, flavonoid, and phenolic compound contents.

Partial biodegradation of XPS was evidenced in the larvae’s frass. A comprehensive characterization of the frass was performed using SEM, HPSEC, FTIR, and TGA techniques, and this revealed a decrease in the molecular mass, changes in the chemical structure of XPS, and the presence of reaction byproducts. Biodegradation results in oxidized XPS oligomers, and we observed the most effective XPS degradation in the frass from smaller larvae fed a diet containing used XPS packaging (frassXPSPC-L1).

The results presented here suggest that the use of *Zophobas atratus* larvae can serve, at minimum, as a sustainable biological pre-treatment step in the management of XPS waste. The partial oxidation and fragmentation observed in this study may facilitate subsequent microbial or enzymatic depolymerization processes, paving the way toward the development of complete biodegradation protocols for polystyrene foams at larger scales. These findings highlight the potential integration of larval biodegradation into circular waste management systems and encourage further investigation into the ecological safety and optimization of this bio-based approach.

The possible difference in the microbiota of the *Z. atratus* larvae’s gut at different larval stages or their ability to adapt to new diets may influence the XPS biodegradation and generation of intermediate products. However, new optimization studies are required to increase the biodegradation of XPS and elucidate the reaction mechanisms involved in the biodegradation process.

## Figures and Tables

**Table 1:** Description of the samples. Larval groups L1 and L2 were fed with a commercial diet, pristine XPS, and post-consumer XPS packaging.

Sample Code	Description
RC	Control sample of commercial diet
RC-L1	Larvae of group L1 fed with commercial feed only
RC-L2	Larvae of group L1 fed with commercial feed only
frassRC-L1	Frass of larvae of group L1 fed with commercial feed only
frassRC-L2	Frass of larvae of group L2 fed with commercial feed only
XPSLP	Control sample of pristine XPS packages
XPSLP-L1	Larvae of group L1 were fed with pristine XPS feed only
XPSLP-L2	Larvae of group L1 were fed with pristine XPS feed only
frassXPSLP-L1	Frass of larvae of group L1 were fed with pristine XPS feed only
frassXPSLP-L2	Frass of larvae of group L2 were fed with pristine XPS feed only
XPSPC	Control sample of post-consumer XPS packages
XPSPC-L1	Larvae of group L1 fed with post-consumer XPS feed only
XPSPC-L2	Larvae of group L2 fed with post-consumer XPS feed only
frassXPSPC-L1	Frass of larvae of group L1 fed with post-consumer XPS feed only
frassXPSPC-L2	Frass of larvae of group L2 fed with post-consumer XPS feed only

**Table 2:** Moisture content, lipids, identification and normalization of fatty acids, phenolic compounds, and flavonoids of *Z. atratus* larvae—Group L2.

Diet	Moisture (%)	Lipids (%)	C16:0	C18:0	C18:1 ω-9	C18:2 ω-6	Phenolics (mg/100g)	Flavonoids (mg/100g)
RC-L2	56.23 ± 1.15	15.30 ± 0.67	37.11 ± 1.31	8.16 ± 0.21	30.61 ± 0.69	22.25 ± 0.69	3.26 ± 0.53	0.012 ± 0.006
XPSLP-L2	67.77 ± 13.30	3.47 ± 0.48	33.44 ± 0.24	10.37 ± 0.09	31.82 ± 0.17	22.25 ± 0.02	5.59 ± 0.47	0.040 ± 0.008
XPSPC-L2	78.12 ± 0.58	2.96 ± 0.75	33.53 ± 0.23	10.31 ± 0.40	33.31 ± 0.23	20.79 ± 0.29	1.72 ± 0.07	0.041 ± 0.019

## Data Availability

The data presented in this study are available on request from the corresponding author. The data are not publicly available due to ongoing complementary studies.

## Supplementary Materials

The following supporting information can be downloaded at: https://www.mdpi.com/article/10.3390/polym17212870/s1, Figure S1: Diets used in the experiment. (a) XPSLP. (b) XPSPC. (c) RC; Figure S2: Larval rearing. (a) Smaller larvae—group L1. (b) Larger larvae—group L2. (c) Shelf with the experiment boxes. (d) Larval cannibalism. (e) *Zophobas atratus* in the larval, pupal, and beetle stages; Figure S3: FTIR spectra of clean XPS packaging (XPSLP); Table S1: Consumption rate, survival rate, and average weight, *Z. atratus* larvae title; Table S2: The number-average molar mass (M_n_), weight-average molar mass (M_w_), Z-average molar mass (M_z_), and polydispersity index (PDI) of XPSLP/XPSPC and larval waste (L1 and L2).
