# Supplementary material for: Biodegradation of Pristine and Post-Consumer Extruded Expanded Polystyrene Packaging by Zophobas atratus Larvae: Influence of the Larval Stage and Physiological Response
Source: Polymers (Basel). 2025 Oct 28;17(21):2870. doi: 10.3390/polym17212870 (PMC12608909; doi:10.3390/polym17212870)
Supplement: Supplementary file 1 [file polymers-17-02870-s001.zip › polymers-3931640-supplementary.pdf]

Biodegradation of Pristine and Post-Consumer Extruded Expanded Polystyrene Packaging by Zophobas atratus Larvae: Influence of the Larval Stage and Physiological Response

Supplementary material

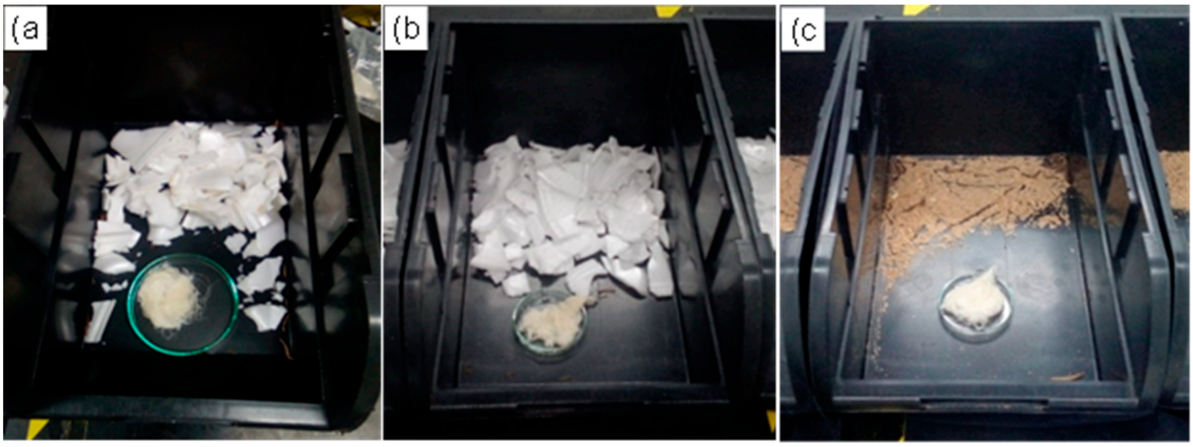

Fig. S1. Diets used in the experiment. (a) XPSLP. (b) XPSPC. (c) RC.

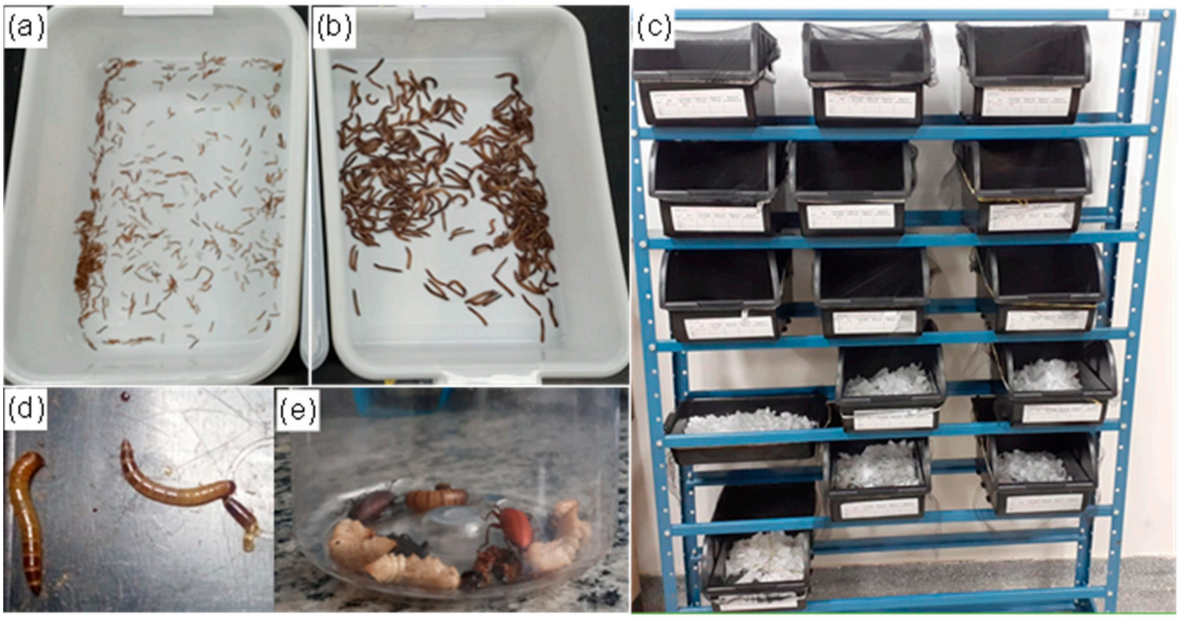

Fig. S2. Larval rearing. (a) Smaller larvae—group L1. (b) Larger larvae—group L2. (c) Shelf with the experiment boxes. (d) Larval cannibalism. (e) *Zophobas atratus* in the larval, pupal, and beetle stages.

Table S1. Consumption rate, survival rate, and average weight, *Z. atratus* larvae

Diet	%Consumption rate each			%Survival rate each			Average larval weight (grams)		
	15 days	30 days	45 days	15 days	30 days	45 days	15 days	30 days	45 days
RC-L1	84.52 ± 0.06	92.20 ± 0.04	94.55 ± 0.04	83.52 ± 7.92	81.32 ± 9.52	80.22 ± 10.60	0.0593 ± 0.0133	0.1251 ± 0.0121	0.2847 ± 0.0114
XPSLP-L1	2.68 ± 0.00	1.20 ± 0.01	0.83 ± 0.00	81.32 ± 3.96	48.35 ± 2.91	17.22 ± 0.63	0.0340 ± 0.0020	0.0513 ± 0.0028	0.0703 ± 0.0068
XPSPC-L1	21.46 ± 0.02	4.78 ± 0.01	1.38 ± 0.01	80.22 ± 9.52	46.52 ± 6.62	17.22 ± 4.16	0.0443 ± 0.0045	0.0673 ± 0.0048	0.1127 ± 0.0286
RC-L2	97.20 ± 0.03	65.20 ± 0.16	40.87 ± 0.04	96.42 ± 1.24	93.19 ± 2.71	89.78 ± 2.69	0.3692 ± 0.0331	0.5032 ± 0.0500	0.6243 ± 0.0508
XPSLP-L2	4.42 ± 0.02	2.36 ± 0.01	0.13 ± 0.00	87.10 ± 3.88	74.19 ± 3.88	64.87 ± 16.70	0.1987 ± 0.0368	0.1981 ± 0.0220	0.2011 ± 0.0201
XPSPC-L2	29.37 ± 0.07	6.57 ± 0.00	3.01 ± 0.01	100.00 ± 0.00	96.39 ± 3.82	89.92 ± 3.35	0.2479 ± 0.0240	0.2560 ± 0.0306	0.2569 ± 0.0254

Table S2. The number-average molar mass (M_n), weight-average molar mass (M_w), Z-average molar mass (M_z), and polydispersity index (PDI) of XPSLP/XPSPC and larval waste (L1 and L2)

Parameter	XPSLP (Da)	frassXPSLP-L1 (Da)	frassXPSLP-L2 (Da)	XPSPC (Da)	frassXPSPC-L1 (Da)	frassXPSPC-L2 (Da)
M_n	128606	115474	122810	130173	124049	114427
M_w	297288	266348	270807	297176	276033	258747
M_z	526386	462497	468900	525558	485562	454218
PDI	2.31	2.31	2.21	2.28	2.23	2.26

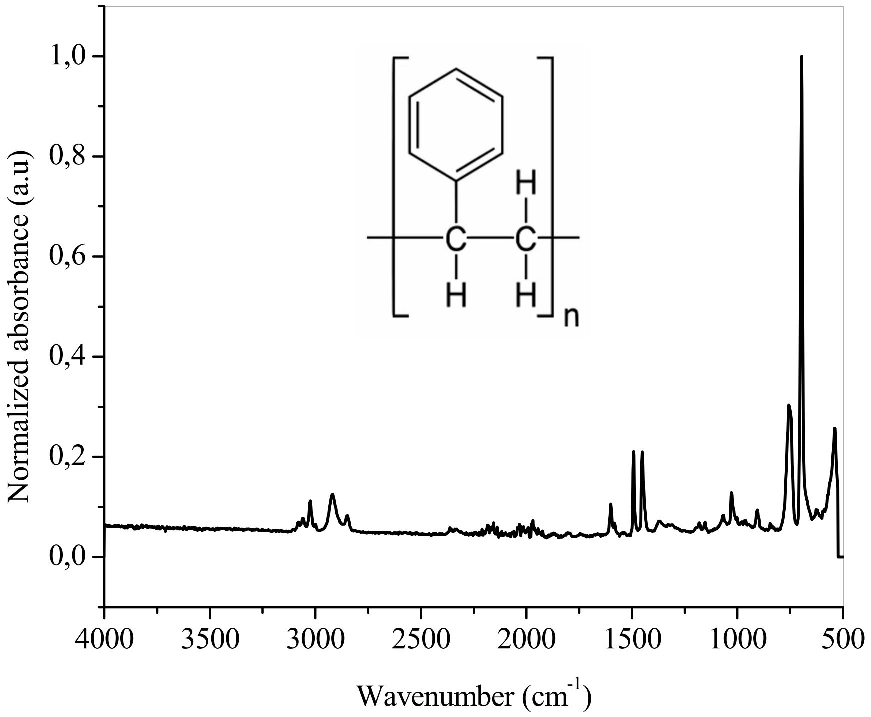

Fig. S3. FTIR spectra of clean XPS packaging (XPSLP)
